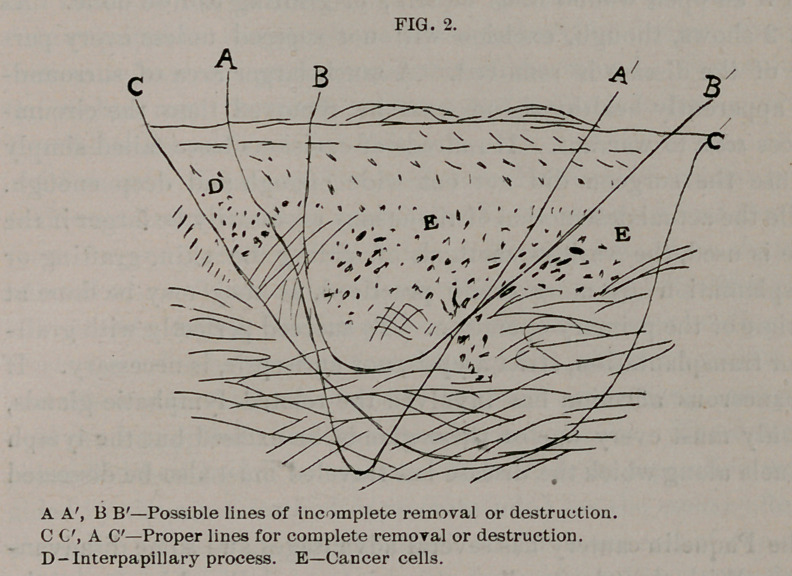# The Practical Treatment of “Cancer” of the Skin

**Published:** 1895-06

**Authors:** M. B. Hutchins

**Affiliations:** Clinical Lecturer on Diseases of the Skin and Syphilis; Demonstrator of Anatomy and Histology, Atlanta Medical College, Atlanta, Ga.


### ATLANTA
### Medical and Surgical Journal.

VOL. XII. JUNE, 1895. No. 4.

LUTHER B. GRANDY, M.D., MANAGING EDITOR. MILLER B. HUTCHINS, M.D., BUSINESS MANAGER.

### *ORIGINAL COMMUNICATIONS.*

### THE PRACTICAL TREATMENT OF "CANCER" OF THE SKIN.

BY M. B. HUTCHINS, M.D.,

Clinical Lecturer on Diseases of the Skin and Syphilis; Demonstrator of Anatomy and Histology, Atlanta Medical College,

ATLANTA, GA.

In my paper read before the State Medical Association last year I gave the diagnostic points in, and the treatment of, the various malignant growths of the skin, a large part of that paper being taken up with epithelioma, as the more frequent of the diseases described. The paper was composed largely of the principles of diagnosis and treatment laid down by the leading authorities.

The present article is intended to present my own experience in the management of cutaneous epithelioma, most of the cases upon which it is based having come under my care since the reading of the paper above referred to.

For the better understanding of the proper principles of treatment of these lesions, I present in Fig. 1 a sketch of a minute sectional portion of a small epitheliomatous lesion of the inner end of the right upper eyelid, as seen under a low power of the microscope. For the sake of clearness the details are drawn as if the specimen were more highly magnified.

FIG. 1.

H—Horny layer. R—Rete.

A—Cancer cells—epithelial down- and outgrowth. B—Interpapillary process of rete.

C—True skin. D—Blood-vessels with inflammation.

At the surface we have the horny layer of the epidermis; next below are the cells of the rete of the epidermis (the lucid and granular layers are not shown). To the right and left of the center appears practically normal epidermis, with one interpapillary process on the left dipping down between the papillæ of the true skin. In the center we have a down-growth of epithelial cells from the epidermis and an infiltration of cells, connected with these, into the tissues of the skin. The rest of the drawing shows the true skin, on the left inflammation as evidenced by the minute, round cells which mark the course of the blood-vessels or obscure them entirely.

In Fig. 2 is shown a rough diagrammatic sketch of an epithelioma, microscopically, with lines which show the limits of successful treatment, and other lines which show incomplete destruction on removal of the disease. If the tissues included between A A′ or between B B′ only are removed, we have left a center from which the disease soon regrows, the wound probably never healing. If, on the other hand, the tissue cut out or destroyed is all within the lines C C′ or A C′, there is no doubt of a cure. The

FIG. 2.

A A′, B B′—Possible lines of incomplete removal or destruction.

C C′, A C′—Proper lines for complete removal or destruction.

D—Interpapillary process. E—Cancer cells.

presumption is that we have taken the disease before there has been time for passage of the cells through the lymph channels, or into the related glands, and this cannot always be known without careful study with the microscope. Usually, however, the clinical appearance is such that we can decide this point, as we must, without the aid of sections.

The methods of treatment which I have employed will be given somewhat in detail. The indications which govern the selection of any method are, the situation of the lesion, the least disfigurement to be obtained, and the extent of the lesion. Besides, the prejudices or fears of the patient must sometimes govern our decision; and, lastly, other things being equal, the convenience of the operator may decide the choice.

The knife is the only method for some cases, as in one of mine where half the upper lip was involved in its whole thickness, necessitating its removal from the median line to a point a fourth of an inch beyond the oral angle. The cut edges were so adapted as to leave a good mouth—union complete in eleven days. Under thorough antisepsis, or, better, asepsis, the knife offers promise of practically no scar, if suturing is possible, while healing begins earlier even if an open wound must be left; or grafting can be done. As Fig. 2 shows, though, excision will not succeed unless every particle of the disease is removed. A much larger area of surrounding, apparently healthy tissue *must* be removed than the circumstances *seem* to warrant. Hundreds of excisions have failed simply because the surgeon did not cut wide enough and deep enough. While the actual destruction of tissue may necessarily be larger if the knife is used, the various methods of sliding the skin, grafting or transplantation are more easily practiced, as they may be done at the time of the primary operation. To succeed perfectly with grafting or transplantation, strict asepsis, not antisepsis, is necessary. If the cancerous affection has involved the related lymphatic glands, not only must every one of these glands be excised but the lymph channels along which the disease has traveled must also be dissected out.

The Paquelin cautery has several advantages and some disadvantages. With the "button" or the point at a dull red heat growths as large as the end of the thumb, or as wide as a half dollar, may easily be destroyed. If the point is at a white heat you will not only have a little blazing of the skin at the edge, but it is likely that there will be such a clean burning of any vessel present that hemorrhage is more likely, but with the dull red heat the destruction is perfect, and the charring usually prevents the slightest bleeding. The Paquelin's advantages lie in the non-necessity of antisepsis or asepsis at the operation (the heat furnishes both) and the fact that in destroying the visible disease you really destroy considerable of the surrounding tissue, or so affect it that any imperceptible offshoots of the disease are also reached. Further, the work can be done much more quickly than with the knife (no bleeding), and an assistant can be dispensed with. In certain situations, as in a case of mine with the affection in the inner canthus, the Paquelin point was chosen because the result with the knife was doubtful, and the use of caustics undesirable. The eyeball was protected with wet cotton, and the lids with adhesive strips. As disadvantages of the Paquelin, we have the fact that we must wait one to two weeks for the eschar to separate before we get the wound to begin healing; also, the idea of red hot metal terrifies some people, though I have had little trouble on this score. The patient can go away for the time required for the separation of the eschar if he desires, returning in time to have the proper treatment of the wound. Separation of the eschar may be aided by the use of a simple oil or ointment. Forcible removal is undesirable.

Of caustics, I have of late used the solid stick of caustic potash nearly exclusively. Any crusts should be removed and the stick (your fingers protected with cotton or forceps) bored into the whole affected area. There is usually produced at once a jelly-like, blackish necrosis, with a whitish-yellow border in the surrounding skin. Parts desired to be protected can be swabbed with dilute acetic acid, and when you think the caustic has been acting long enough the acid can be applied directly to the burned tissues. The destroyed tissue had best be left to separate of itself, or its separation may be aided by the same method as recommended for the eschar after the Paquelin. I used the caustic potash on the inner end of the upper eyelid after clipping out the lesion from which Fig. 1 was drawn, the eyeball being protected with dilute acetic acid. This caustic is often useful and curative in cases where a part of the wound, after other methods, refuses to heal, showing incomplete destruction of the disease. Marsden's paste (acid. arsenios. pulv. acaciæ, aa gr. xx., aq. q. s. ut ft. paste) I have not used lately, because it must be left on from twelve to forty-eight hours, with just that many hours of more or less pain (morphine, of course, mollifies this), and because I have thought I was able to get as good results with quicker methods. Advantages claimed for this method are, its destroying only diseased tissue, leaving less of a scar, and I believe it is also claimed that the destruction of the cancerous disease extends far wider than one would think of cutting (Robinson's personal statement). Marsden's paste will suit an open ulcerated lesion, but its work is unsatisfactory upon the papillary form. I have no experience with combined excision and cauterization, though I believe it would be exactly right in certain cases. Electrolysis is slow, and as there is stimulation around (though destruction in the course of) the needle, I am a little afraid of it. I used the needle to destroy the last recurrent papule in a very obstinate case. I have seen every one of the methods which I have described followed by return of the disease, and I have seen every one of them succeed in curing the disease, fortunately the latter condition prevailing in the majority. The returns simply showed incomplete treatment, and serve to impress what should be the guiding rule of those who treat this class of disease: *Radically destroy every particle of cancerous disease. If not done the first time, be sure there is no chance of a second failure.* Of course we cannot always tell whether we have followed the rule or can follow it. A few weeks or months after the treatment may reflect more light upon this subject. There are cases which are untreatable, or which at best can only be treated palliatively. It remains to be seen what experiments with nuclein, hypodermically and by the stomach, as a destroyer of the yet undiscovered cancer germ will result in.

After-treatment is, of course, simply the treatment of a wound. If sutures and first intention are practicable, it is brief and easy. Grafting is more trouble, but the results justify the trouble. An open wound requires more time and variety of treatment. Given a healthy wound, cleanliness and simple bismuth without other dresssing may suffice. If there is pus, antiseptic solutions followed by iodoform and iodoform gauze five to ten per cent. are proper. I believe iodoform interferes with the healing where there is no pus for it to neutralize, and its odor is decidedly objectionable. Unless the wound is large (most of my cases have been of the face), I do not use bandages. If the powder used does not form a sufficient crust, five per cent. iodoform gauze will usually stick; if not, a dressing may be held all around its edges with collodion. Unless the presence of pus or contraction of dressings renders frequent dressings necessary, I think it better not to change the dressings every day, as the new epidermis is liable to injury. Sometimes, however, frequent dressings give a much less contracted scar.

The ideal treatment of these wounds is absolute asepsis, and the direct application of a piece of antiseptic rubber tissue. Oils and ointments I have not used, except for the removal of crusts, if such formed. As wound stimulants I have used four per cent. nitrate silver solution, or the stick, or liquefied carbolic acid as indicated.

Of anæsthetics ether was used where necessary; chloroform if the operation was about the mouth, beginning with ether and finishing with chloroform. As a local anæsthetic I use a four per cent. solution of cocaine hypodermically, first always giving the patients half an ounce to an ounce of whiskey, as the whiskey so administered certainly acts as an antidote to the bad effects of cocaine. I have once used a one per cent. solution of cocaine, but did not find it satisfactory. The cocaine solution may be applied directly to the ulcerated surface in caustic or cautery treatment if ulceration exists, but it is doubtful if the absorption is sufficient for anæsthesia. The proper method is hypodermically, and possibly endermically. The needle is best pushed into the skin at the edge of the lesion and through to the opposite side (for "burning" methods), gradually withdrawing the needle as the injection is made. Then, keeping the needle in the same skin puncture, it should be pushed in other directions, and so on until the whole area to be treated is anæsthetized. The same plan, following the proposed incisions, is proper in knife cases. The advantage of a single skin puncture is that the chief pain of the insertion of the needle comes from the skin. I have made it a rule to use a fresh solution of cocaine, as it does not keep well, and various expedients to make it keep seem to add to the pain of the injection.

330 *Equitable Building.*

---

### A CASE OF SOFT CHANCRE OF THE EYELID.

Dr. Ole B. Bull, of Christiania, referring to a case of hard chancre of the eyelid, reported by Dr. Holth (*Norsk Magazin for Lægevidenskaben*, p. 383, 1894), where he thought transmission to have taken place from kissing, records a case of soft chancre of the right upper eyelid in a patient with a soft chancre of the penis, from which it was transferred to the eyelid by rubbing his eye with his finger on account of a foreign body. As this localization is much rarer in Scandinavia than in France, it proves that kissing is more in vogue in the south than in the north.—*Journ. Cut. and Gen. Ur. Dis.*, March, 1895.

## Figures and Tables

**Fig. 1. f1:**
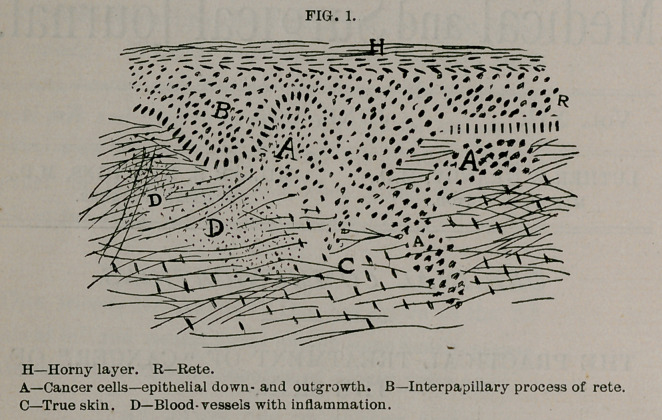


**Fig. 2. f2:**